# Supplementary material for: Saccharomyces boulardii (CNCM I-745) improves intestinal damage in sepsis by remodeling intestinal flora structure
Source: Front Cell Infect Microbiol. 2025 Jul 31;15:1602792. doi: 10.3389/fcimb.2025.1602792 (PMC12350329; doi:10.3389/fcimb.2025.1602792)
Supplement: Supplementary file 1 [file DataSheet1.zip › summary/summary/8_Advanced_analyse/2_PICRUSt2_result/KEGG/Group.DW4_vs_DW0_vs_CW4_vs_CW0_vs_BW4_vs_BW0_vs_AW4_vs_AW0/PICRUSt2_KEGG_level3_Group.DW4_vs_DW0_vs_CW4_vs_CW0_vs_BW4_vs_BW0_vs_AW4_vs_AW0_diff.pdf]

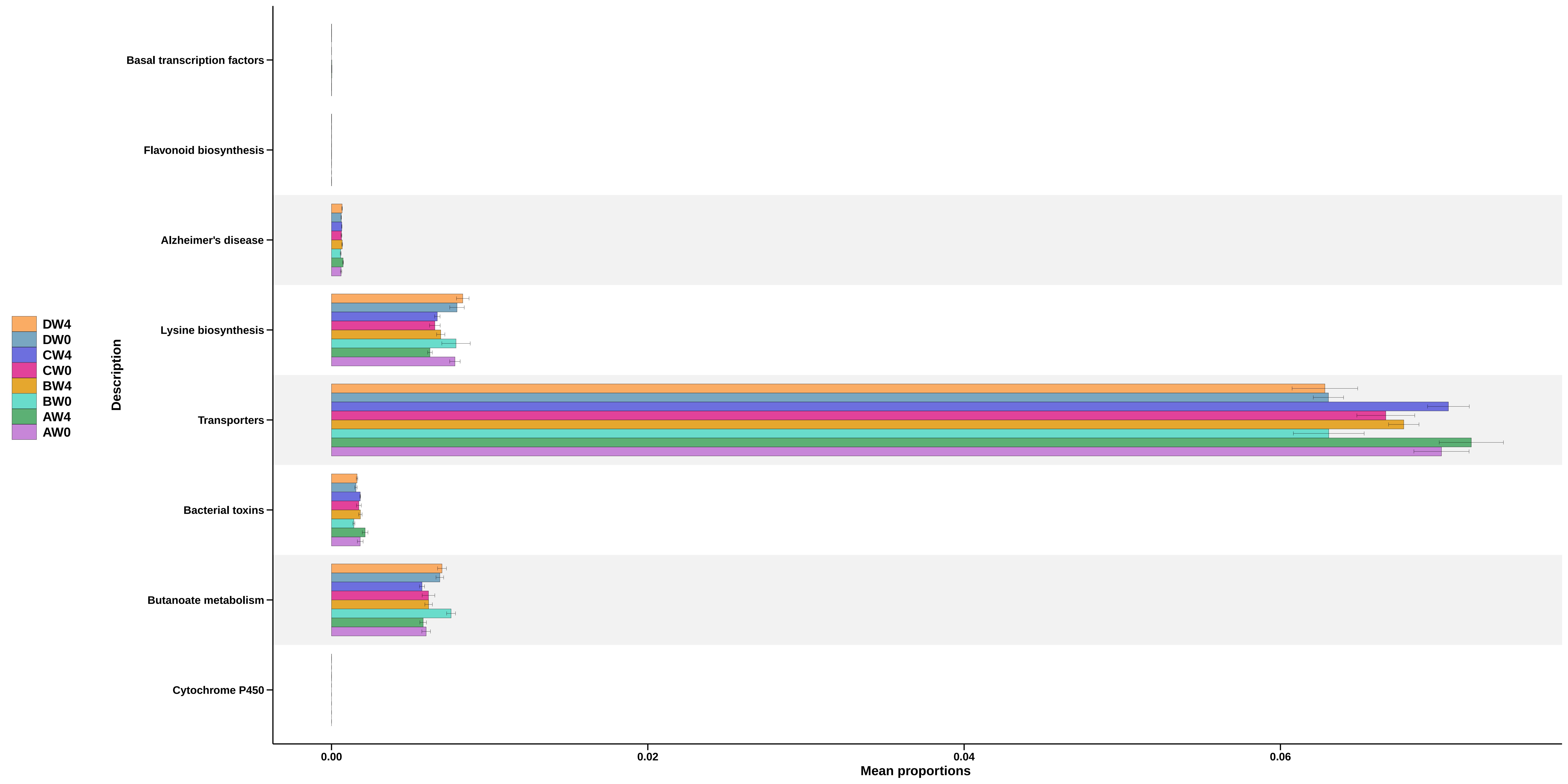
DW4
DW0
CW4
CW0
BW4
BW0
AW4
AW0
Description
Basal transcription factors
Flavonoid biosynthesis
Alzheimer's disease
Lysine biosynthesis
Transporters
Bacterial toxins
Butanoate metabolism
Cytochrome P450
0.00
0.02
0.04
0.06
Mean proportions
